# The Neural Correlates of Dreaming Have Not Been Identified Yet. Commentary on “The Neural Correlates of Dreaming. Nat Neurosci. 2017”

**DOI:** 10.3389/fnins.2020.585470

**Published:** 2020-10-30

**Authors:** Perrine Marie Ruby

**Affiliations:** Lyon Neuroscience Research Center, CNRS UMR 5292 - INSERM U1028 - Lyon 1 University, Lyon, France

**Keywords:** dream recall, brain, EEG, inconsistent, neurophysiological marker of dreaming

Dreaming is a special topic to investigate with a scientific approach. Its main characteristic is to be difficult to access and to observe which prevents many experimental approaches from being possible or successful (e.g., Scarpelli and De Gennaro, 2017; Plailly et al., 2019). Indeed, no reliable (neuro)physiological correlates of dreaming have been identified yet which means that one cannot know whether a sleeper is dreaming or not while she/he is sleeping [except in the peculiar and rare case of lucid dreaming, Vallat and Ruby (2019)]. Our only access to dreaming is still dream reports, which are made a posteriori, during wake and which are possibly partial and/or modified by the waking consciousness. This state of fact seriously hampers the cognitive neuroscience of dreaming and especially the identification of the neural correlates of dreaming since one cannot measure a cerebral activity during dreamful sleep versus dreamless sleep. However, in 2017, the Siclari et al. article published in Nature Neuroscience (2017) claimed that they identified “The neural correlates of dreaming.” The visibility of this article had had and still has an important impact on the research on sleep, dreaming and memory since it is considered as a golden standard, a reference. It cannot be ignored or a reviewer asks you to quote it and the spreading of its message induces the idea that “The neural correlates of dreaming” have been identified. Taking into account the available scientific data, we argue below that it is not the case. We believe that this commentary is necessary to prevent undue consequences on future research, such as the spreading of a message not supported by the available scientific data and the drastically diminished chance to obtain funding to investigate/uncover the neural correlates of dreaming. Are detailed below the theoretical and methodological concerns identified reading the Siclari et al. article published in Nature Neuroscience (2017) and preventing one from concluding that their data unveiled “The neural correlates of dreaming.”

**1. The title “The neural correlates of dreaming” is misleading : it is not faithful to the paradigm used by the authors**.. The authors analyzed the electroencephalogram (EEG) in the sleep preceding a dream report (Figure 1). The results obtained using this paradigm (let's call it the serial-awakening paradigm) cannot be interpreted as the neural correlates of dreaming, they show some cerebral activity predicting dream recall at best. To be able to claim that they identified the electrophysiological correlates of dreaming, the authors should have recorded the EEG during sleep associated with dreams versus dreamless sleep. This is however currently not possible because no reliable (neuro)physiological marker of dreaming has been identified yet.

**Figure 1:**
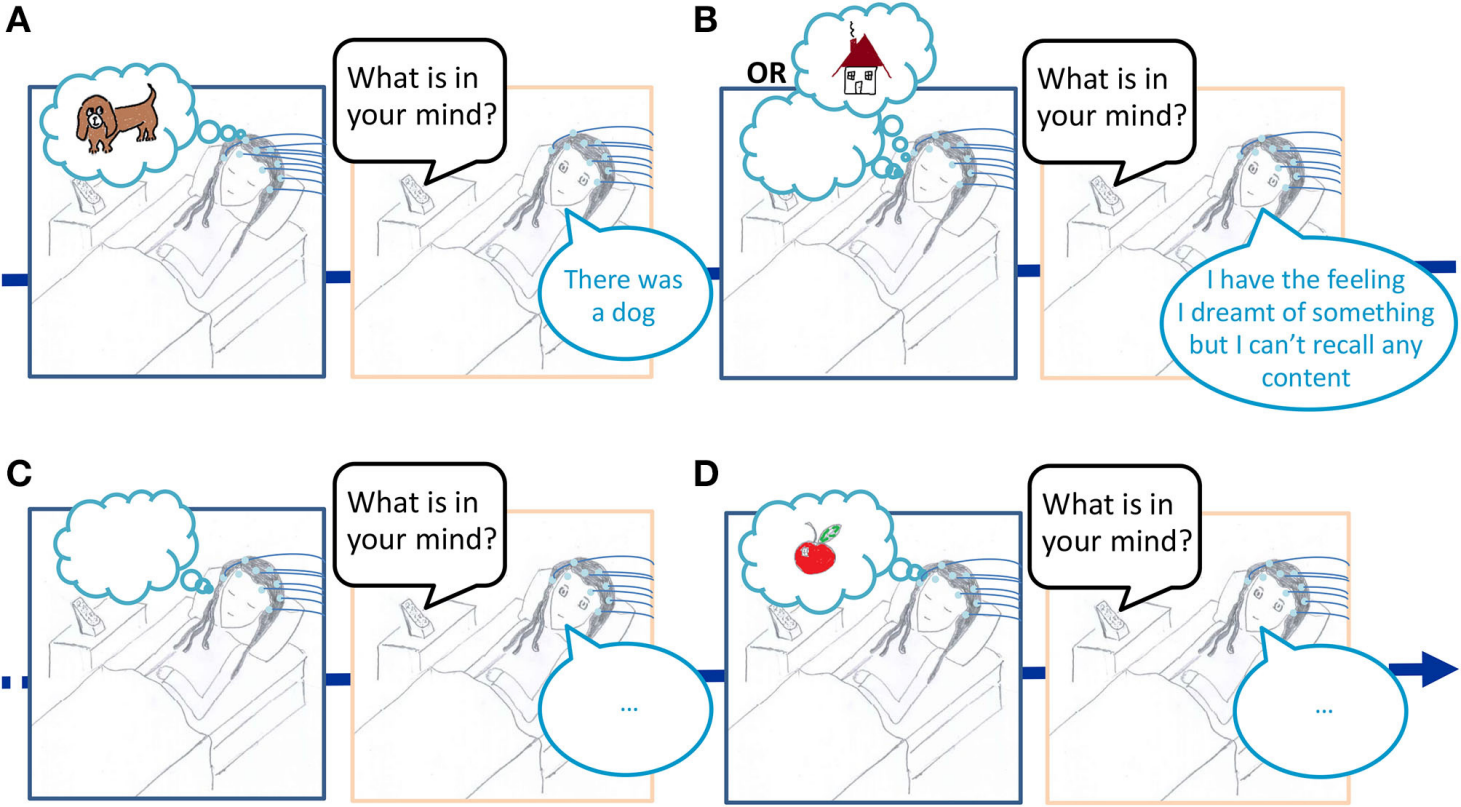
Illustration of the serial-awakening paradigm used in the article “The neural correlates of dreaming” Nat Neurosci 2017. While a participant is sleeping in the lab with EEG electrodes on the scalp, he/she is awakened several times to report what was in his/her mind just before awakening. The EEG signal during sleep is then classified either as “preceding a dream report,” **(A)** “preceding a white dream report,” **(B)** or “preceding no dream report.” **(C,D)** The comparison of the EEG pattern for the sleep “preceding a dream report” vs. “preceding no dream report” is then interpreted as the neural correlates of dreaming in the article “The neural correlates of dreaming” Nat Neurosci. 2017.

**2. The results of the studies using the serial-awakening paradigm are difficult to interpret given the limitations of the paradigm**. Indeed when one recalls no dream at awakening it can be because the preceding sleep was dreamless (Figure 1C) or because a dream memory has been forgotten (Figure 1D). But the case presented in Figure 1D (the forgotten dream), is not taken into account in the article “The neural correlates of dreaming,” since the authors considered that no dream reports equals no dream activity in the preceding sleep. This is problematic because, for the EEG classified as associated with no dream report in their study, a dreaming activity might have been concomitant (it is well-known that a stimulus in the environment during the day can trigger the recall of a dream which had not been recalled in the morning at the time of awakening), and the moment when the memory of the dream had vanished is unknown. As a consequence, contrasting the EEG signals during sleep classified as “preceding a dream report” vs. “preceding no dream report,” cannot reveal the neural correlates of dreaming, but may only give some limited insight into the electrophysiological activity preceding a dream recall. The limitation comes from the fact that (1) when a dream is recalled, one doesn't know when it precisely happened and how long it lasted, as a consequence the analyzed sleep EEG may or may not have been concomitant with the dream experience recalled, (2) when no dreams are recalled, one cannot know whether no dream were experienced or whether a dream was experienced and then forgotten (and for the forgotten dreams the moment when the forgetting process occurs is also unknown), it thus entails that in the condition “no dream recall” in Siclari et al. (2017) the analyzed sleep EEG may mix both dreamful and dreamless sleep in unknown proportions. Such limitations should result in varying and inconsistent results across repeated studies, and it is indeed what has been observed so far (see point 4).

**3. The serial-awakening paradigm used in the “The neural correlates of dreaming” article has been used in 14 previous studies (see Table 1) but none of them are quoted**. Given that those studies are not cited, the authors did not discuss their results in their scientific context. The non quotation of these studies also gives the wrong impression to the reader that the authors' team was the first one to issue the idea of this paradigm and diminishes the chances to get appropriate/expert reviewers.

**Table 1:** Review of the studies that investigated the pre-awakening sleep EEG spectral power associated with the presence or absence of a dream report after awakening.

	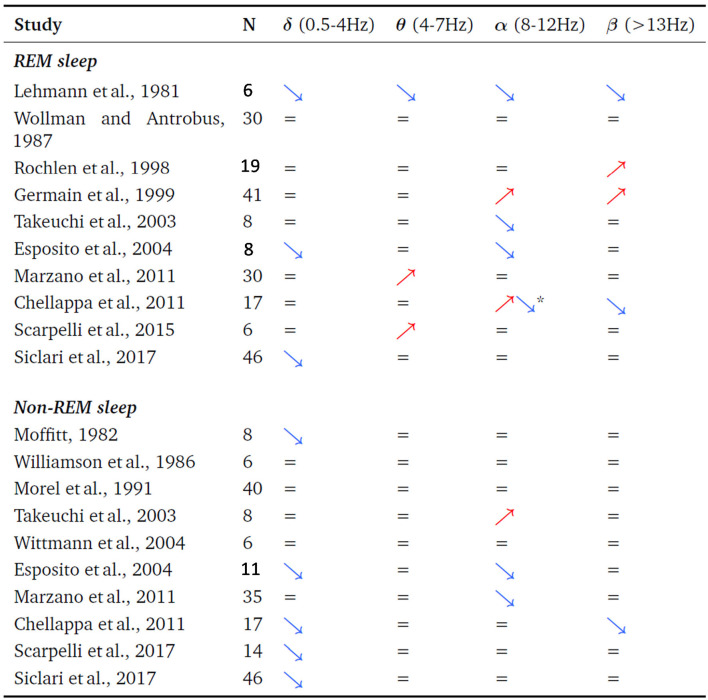	

↗, the EEG spectral power is increased in this frequency band when subjects recalled a dream compared to when they did not recall one. ↘, the EEG spectral power is decreased in this frequency band when subjects recalled a dream compared to when they did not recall one. =, no significant EEG spectral power difference in this frequency band were observed, between the two conditions. *Higher occipital alpha, decreased frontal alpha. N = number of participants.

**4. All the previous EEG studies which used the serial-awakening paradigm resulted in different and inconsistent results (see Table 1)**. The authors did not confront their results and methods to previous ones in the field and thus did not engage in the necessary critical review of previous work, and their own. Only trying to clarify systematic and unsystematic effects, and proposing possible explaining factors for inconstancies between studies could have bring some light and enduring progress in the field of research investigating the neural correlates of dreaming. Also, not quoting those studies, the authors did not take the chance to explain why they believe that their results are more to be trusted than all the other previous different ones, and leaves the false impression to the reader that the results presented are reliable. Note also that none of the studies presented in Table 1 claimed to have discovered the neural correlates of dreaming, they all interpreted their results in terms of dream recall.

**5. Coherently with this literature (Table 1), post-2017 studies also yielded various and inconsistent results**. Some studies partially replicated the Siclari et al. (2017) results (D'Atri et al., 2019; Scarpelli et al., 2019, 2020) while some didn't. The recent Wong et al. (2019) study failed to observe any significant difference between the “dream recall” vs. “no dream recall” condition. Using the same serial awakening paradigm, their blinded analyses disconfirmed markers of dreaming consciousness in EEG spectral power. The Wong et al. study is a supplementary argument confirming that the serial awakening paradigm produces unreliable results. Unfortunately, at the end, the scientific benefit of invalidating the results presented in “The neural correlates of dreaming” is scarce because previous work already demonstrated the low reliability of the results obtained with this paradigm and also because the visibility of the Wong et al. article (as all the other ones in Table 1) is far lower than the visibility of the Siclari et al. article in Nature Neuroscience. As a consequence, it is to be expected that the Wong et al. article won't be able to change the message spread in the scientific community, and the false belief (i.e., the neural correlates of dreaming have been identified) contradicting what has been observed for decades by several teams will most probably remain. This commentary aims at diminishing the high risk that biased or false beliefs about the neural correlates of dreaming expand and last, and induce further research programs which are doomed to fail. However, as the Wong et al. article, this commentary has little chances to change beliefs as much as necessary, for the same reason again, because Frontiers has a far lower visibility than Nature Neuroscience. We tried to submit this commentary to the journal who published the Siclari et al. article. However, the Nature Neuroscience Editor, Jean Mary Zarate, did not find appropriate to send this comment to reviewers (in July 2020). In other words, the journal who published the article “The neural correlates of dreaming” is indifferent to the fact that this article does not fulfill some of the basic requirements for good science (i.e., quoting and discussing previous results in the field and not over/misinterpreting the results). Such state of facts seriously questions the possibility of fair and sound Science in a system which refuses to face its mistakes.

**6. The results reported in “The neural correlates of dreaming” article are partially consistent with previous neuropsychological and neuroimaging results on dream recall but none of them are cited (Murri et al., 1984; Solms, 1997; Bischoff and Bassetti, 2004; Eichenlaub et al., 2014)**. Again, some studies directly related to the topic addressed in the Siclari et al. article are not quoted by the authors. And, contrary to the studies presented in Table 1, these studies, comparing the brain of persons with a high dream recall frequency (HR) to the brains of persons with a low dream recall frequency (LR), resulted in quite consistent results even while using different techniques (neuropsychology and neuroimaging in healthy subjects). They highlighted an increased activity in the temporo-parietal junction (TPJ) and medial prefrontal cortex in HR as compared to LR during sleep and wakefulness. The TPJ, known to be involved in episodic memory (Wagner et al., 2005), is part of the posterior hot zone described in Siclari et al., whose activity increases before awakening when the awakening is followed by a dream recall. It is known for a long time now that a lesion in this brain region is associated with a cessation of dream reports (Murri et al., 1984; Solms, 1997; Bischoff and Bassetti, 2004). All these results point to a role of the TPJ/posterior-hot-zone in dream recall.

**7. Based on the phenomenology of dream content [predominantly vivid sensory-motor experience lived as real and involving social interactions, Vallat et al. (2017)], the cerebral correlates of dreaming should involve sensory-motor and limbic corticies**. These brain regions are activated in a phasic way during sleep, at least during REM sleep (Hong et al., 2009; Miyauchi et al., 2009) but their association with dream content has not been demonstrated yet (Ruby, 2011).

In conclusion, the characteristics of the paradigm used in the study “The neural correlates of dreaming. Nat Neurosci. 2017” and the previous uncited studies on the subject [either using the same paradigm (the serial awakening paradigm), or on the same topic (dream recall)], show that the results of Siclari et al. (2017) cannot be interpreted as the neural correlates of dreaming, but at best as the neural correlates of dream recall. In addition, the large variability of the results obtained with the serial awakening paradigm and the recent failed replication of “The neural correlates of dreaming. Nat Neurosci. 2017” question the reliability of Siclari et al. (2017) results. The primary aim of this commentary is to provide the readers with all the scientific arguments available on the subject and to prevent the spreading of a not scientifically demonstrated message.

At a larger scale, the concerns raised in this commentary point to the worrying limitations of the current international scientific publications system whose top one priority seems to be drifting more and more away from scientific soundness, reliability and originality. The case presented here also highlights the always necessary need of theoretical framework and thinking to inform and ground experimental work (e.g., Valli and Revonsuo, 2009; Valli, 2011; Windt, 2015; Roenneberg, 2019). Experimental science may get lost without conceptual science to guide interpretation and prediction and to provide tools to disentangle between several possible mechanisms or interpretations. This is certainly the case in the field investigating the cerebral correlates of dreaming. We are currently stuck at the experimental level because no proper ways to investigate the dreaming brain are available. Only, new ideas, concepts or methods may allow scientists to overcome the challenges that dreams are throwing at us for hundreds of years.

## Author Contributions

The author confirms being the sole contributor of this work and has approved it for publication.

## Conflict of Interest

The author declares that the research was conducted in the absence of any commercial or financial relationships that could be construed as a potential conflict of interest.
